# Profile pressure between hypertensive with and without diabetes

**DOI:** 10.1186/1758-5996-7-S1-A132

**Published:** 2015-11-11

**Authors:** Andréa Cristina de Sousa, Haroldo Silva de Souza, Thiago Oliveira Costa, Marcos Paulo Marinho Montelo, Ana Carolina Arantes, Ymara Cássiao Luciana Araúj, Rafaela Bernardes Rodrigues, Simone Dias Souza de Oliveira, Humberto Graner Moreira, Dalma Alves Pereira, Camila Ferreira de Oliveira, Thiago Sousa Veiga Jardim, Maicon Borges Euzébio, Weimar Kunz Sebba Barroso de Souza, Paulo Cesar Brandão Veiga Jardim, Ana Luiza Lima Sousa

**Affiliations:** 1 Universidade Federal de Goiás, Goiânia, Brazil

## Background

The lack of control of diabetes and hypertension contributes to the high rates of morbidity and mortality from cardiovascular disease. Better blood pressure control can effectively reduce such outcomes and reduce micro and macrovascular complications in patients with these comorbidities.

## Objective

To analyze the control of hypertension in a follow-up cohort of ten yrs. and compare the blood pressure control rates among hypertensive patients with and without diabetes. Materials and Methods: a cross-sectional study of historical cohort of hypertensive/diabetic (GDM) and nondiabetic (GHAS) in regular treatment for at least 10 yrs. in special service for compliance with hypertension. Initial assessment of the cohort in 2004; used variables: gender, race, age, physical inactivity, alcohol consumption, smoking, amount of drugs, levels and control blood pressure, weight and height. For the control of blood pressure among nondiabetic hypertensive patients were considered values <140/90 mmHg and for hypertensive diabetic values <130/80mmHg. Association analysis of variables with chi-square test and means were compared with Student's t-test, with 5% significance. Project approved by the ethics committee.

## Results

The study included 126 patients (GDM 69; GHAS 57), with average length of treatment in 2004 of 6.8 yrs. Females were predominated in both groups (GDM 81.2%; GHAS 77.2%) and also white race (62.5%). The average age of the cohort was 57.7 yrs. (±9.3; 59.3 56,1--95%). The overall mean BMI was 28.9 kg/m2; for GDM average was 30.8 kg/m2 (29.6 to 32.2 95% CI) and the GHAS 26.4 kg/m2 (25.4 to 27.5 95% CI) p <0.001. The overall mean systolic blood pressure was 134.6 mm Hg (95% CI 131.1 to 140.2), and diastolic pressure was 84,1mmHg (86.4 81,7--95%); no significant difference between groups. The pressure control was unsatisfactory for 68.9% of hypertensive patients with diabetes and hypertension 31.1% without diabetes (p <0.001). The numbers of antihypertensive drugs prescribed was similar between groups; there was no association between the amount of medication and blood pressure control.

## Conclusion

The presence of diabetes as comorbidity reflected in worse hypertension control rates, even with amount of antihypertensive medication similar. Diabetic hypertensive patients should receive more effective control actions in order to reduce cardiovascular outcomes because their blood pressure goals are stricter.

